# A Modular and Expandable Ecosystem for Metabolomics Data Annotation in R

**DOI:** 10.3390/metabo12020173

**Published:** 2022-02-11

**Authors:** Johannes Rainer, Andrea Vicini, Liesa Salzer, Jan Stanstrup, Josep M. Badia, Steffen Neumann, Michael A. Stravs, Vinicius Verri Hernandes, Laurent Gatto, Sebastian Gibb, Michael Witting

**Affiliations:** 1 Institute for Biomedicine (Affiliated to the University of Lübeck), Eurac Research, 39100 Bozen, Italy; andrea.vicini@eurac.edu (A.V.); vinicius.verri@eurac.edu (V.V.H.); 2 Research Unit Analytical BioGeoChemistry, Helmholtz Zentrum München, 85764 Neuherberg, Germany; liesa.salzer@helmholtz-muenchen.de; 3 Department of Nutrition, Exercise and Sports, University of Copenhagen, 1985 Frederiksberg, Denmark; jst@nexs.ku.dk; 4 Department of Electronic Engineering & IISPV, Universitat Rovira i Virgili, 43007 Tarragona, Spain; josepmaria.badia@urv.cat; 5 CIBER de Diabetes y Enfermedades Metabólicas Asociadas (CIBERDEM), Instituto de Salud Carlos III, 28029 Madrid, Spain; 6 Leibniz Institute of Plant Biochemistry, Bioinformatics and Scientific Data, 06120 Halle, Germany; sneumann@ipb-halle.de; 7 German Centre for Integrative Biodiversity Research (iDiv) Halle-Jena-Leipzig, 04103 Leipzig, Germany; 8 Department of Environmental Chemistry, Eawag, Swiss Federal Institute of Aquatic Science and Technology, 8600 Dübendorf, Switzerland; michael.stravs@eawag.ch; 9 Institute of Molecular Systems Biology, ETH Zürich, 8093 Zürich, Switzerland; 10 School of Medicine and Surgery, Università degli Studi di Milano-Bicocca, 20854 Vedano al Lambro, Italy; 11 Computational Biology and Bioinformatics Unit, de Duve Institute, Université Catholique de Louvain, 1200 Brussels, Belgium; laurent.gatto@uclouvain.be; 12 Department of Anesthesiology and Intensive Care, University Medicine Greifswald, 17475 Greifswald, Germany; mail@sebastiangibb.de; 13 Metabolomics and Proteomics Core, Helmholtz Zentrum München, 85764 Neuherberg, Germany; michael.witting@helmholtz-muenchen.de; 14 Chair of Analytical Food Chemistry, TUM School of Life Sciences, Technical University of Munich, 85354 Freising-Weihenstephan, Germany

**Keywords:** metabolomics, untargeted analysis, annotation, R programming, small-compound databases, reproducible research

## Abstract

Liquid chromatography-mass spectrometry (LC-MS)-based untargeted metabolomics experiments have become increasingly popular because of the wide range of metabolites that can be analyzed and the possibility to measure novel compounds. LC-MS instrumentation and analysis conditions can differ substantially among laboratories and experiments, thus resulting in non-standardized datasets demanding customized annotation workflows. We present an ecosystem of R packages, centered around the *MetaboCoreUtils*, *MetaboAnnotation* and *CompoundDb* packages that together provide a modular infrastructure for the annotation of untargeted metabolomics data. Initial annotation can be performed based on MS^1^ properties such as *m/z* and retention times, followed by an MS^2^-based annotation in which experimental fragment spectra are compared against a reference library. Such reference databases can be created and managed with the *CompoundDb* package. The ecosystem supports data from a variety of formats, including, but not limited to, MSP, MGF, mzML, mzXML, netCDF as well as MassBank text files and SQL databases. Through its highly customizable functionality, the presented infrastructure allows to build reproducible annotation workflows tailored for and adapted to most untargeted LC-MS-based datasets. All core functionality, which supports base R data types, is exported, also facilitating its re-use in other R packages. Finally, all packages are thoroughly unit-tested and documented and are available on GitHub and through Bioconductor.

## 1. Introduction

Metabolomics studies small molecules that are substrates, products, or intermediates of metabolic reactions in a biological organism or system. One of the major analytical technologies for the analysis of the metabolome is liquid chromatography-mass spectrometry (LC-MS). LC-MS can be employed in targeted mode, analyzing a pre-selected subset of metabolites and/or metabolite classes or untargeted mode aiming to detect as many metabolites as possible. Similar analyses are carried out in environmental research or exposomics. This type of analysis yields features, defined as mass-to-charge-ratios (*m/z*)-retention time (RT) pairs, their corresponding intensity or peak area in the respective samples and potentially one or more associated fragmentation spectra. These features need to be assigned to metabolite entities in order to make sense of metabolomics data, a process called metabolite annotation.

Different levels of annotation and identification have been defined by the Metabolomics Society and others [1,2]. Highest level identifications are achieved by comparing detected features against chemical reference standards measured in the same laboratory with the same analytical methods (normally referred to as in-house database). Since the availability of reference standards is limited, other ways of initial annotation are required, e.g., using public available reference spectra.

Several software tools for metabolite annotation and identification are available [3], ranging from simple accurate mass search to library matching and in silico tools such as CSI:FingerID [4,5]. In the programming and statistical environment R, many software packages for metabolomics data annotation and identification already exist [6]. Among more recently developed R tools are *MetaboAnalystR* 2.0 [7], *patRoon* [8] and *metID* [9]. *MetaboAnalystR* integrates the mummichog algorithm [10] and hence allows users to perform a functional annotation of untargeted metabolomics data. *patRoon* builds upon a large set of software packages and provides automated annotation workflows specifically designed for environmental samples. *metID* is an R package that, by exporting a single function, allows users to perform MS^1^- and MS^2^-based annotations against user-supplied reference databases of *m/z* values, retention times and MS^2^ spectra. The *msPurity* [11] R package also provides a function for spectral matching of query MS^2^ spectra against a reference library. All these packages, similar to most others reviewed in [6], were designed to be used as standalone applications. Most packages lack modularity or the possibility to re-use their core functionality in other packages or workflows without extensive use of glue-code or wrappers to convert between the used data objects. Even more importantly, most packages implement their own routines for data import and MS data handling as well as their own versions of established spectra similarity scores. Furthermore, these are in most cases not exported and are in some cases not covered with unit tests to ensure their correctness. Another common problem is that many R packages are not maintained after their initial publication or are only infrequently updated. From the 39 R packages listed in [6] that provide some functionality for MS annotation and identification, 20 were not updated for more than 2 years, and three have been removed or deprecated. To guarantee reproducible analysis workflows, software maintenance and active development is however essential.

To overcome these issues, we developed a modular ecosystem of R packages, *MetaboCoreUtils*, *MetaboAnnotation*, *MsCoreUtils*, *Spectra*, *MsBackendMassbank*, *MsBackendMgf*, *MsBackendMsp* and *CompoundDb* that together provide a comprehensive infrastructure for the annotation and identification of features from untargeted LC-MS and LC-MS/MS experiments using established methods. Importantly, in addition to the more end-user-oriented functions, reference implementations for a large set of commonly used algorithms are exported, and all functions are extensively documented and come with a comprehensive set of unit tests.

## 2. Results

### 2.1. Package Ecosystem

We implemented a set of R packages that together provide a customizable infrastructure for the annotation and identification of features from untargeted metabolomics or small-compound MS experiments. The infrastructure provides both high-level functions specifically designed for users less experienced with bioinformatic tools as well as low-level core functionality that can be easily integrated and re-used also in other R software packages.

#### 2.1.1. Overview and Architecture

**Figure 1:**
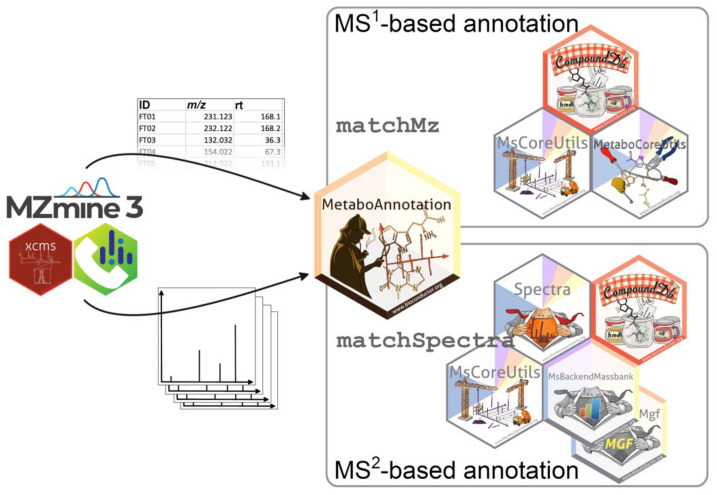
R-package ecosystem for MS^1^- and MS^2^-based annotations. Functionality from the various R packages is combined for specific annotation tasks. The *MetaboAnnotation* package represents the main interface to the end user, while other packages such as *MsCoreUtils*, *MetaboCoreUtils* or *Spectra* provide the base functionality, which can also be easily integrated into other R packages or R-based workflows. A variety of input and output formats are supported, also enabling integration with other analysis software.

This ecosystem comprises the R packages *MetaboAnnotation*, *Spectra*, *MsBackendMassbank*, *CompoundDb*, *MetaboCoreUtils* and *MsCoreUtils* that are maintained under the umbrella of the RforMassSpectrometry initiative (https://www.rformassspectrometry.org, accessed on 4 February 2022). Core functionality such as similarity scores, or other low-level functions were implemented in the *MetaboCoreUtils* and *MsCoreUtils* packages, while the more user-oriented functions were implemented in the *MetaboAnnotation* package. The former packages also allow for re-use of the core functionality in other R packages since the provided functions do not depend on specific data or result object classes. The *MsBackendMgf*, *MsBackendMsp*, *MsBackendMassbank* and *CompoundDb* packages provide access to annotation resources and reference libraries for MS^1^ and MS^2^ annotation. The functionality from all these packages is well integrated with other Bioconductor R packages such as *xcms*, from which pre-processing results can be directly used as the starting point for the annotation workflow. An overview of the various R packages of the presented package ecosystem and a graphical description of which packages are used for the different annotation strategies is provided in Figure 1.

#### 2.1.2. Development and Maintenance Processes

To guarantee a high quality and reliability of the code base and to reduce the number of potential software bugs, a code review from at least one of the experienced core package developers from the RforMassSpectrometry initiative is required for any functionality added to these packages. This extensive code review also ensures a stable code base even if developers would leave the project. Following the development strategy from Bioconductor, all functions are extensively documented, and examples are being provided. To ensure the validity of the packages’ methodology even after future developments or changes in its dependencies or R itself, extensive unit tests are implemented for all functions, which also include comparisons of their results against results from reference implementations, if available. All packages are part of the Bioconductor project guaranteeing reliability, availability, and longtime support. While all the present code was developed by the authors (working at several different institutions), contributions from the community in the form of Github pull requests to the main package repositories are highly welcome.

### 2.2. Utility Functions

Several tasks are recurring in metabolomics data analysis workflows such as calculation of *m/z* from exact masses or handling and formatting of chemical formulas. We implemented utility functions for such tasks in the *MetaboCoreUtils* package. In particular, the package provides functions for handling chemical formulas, for conversion of exact masses to *m/z* and back and for working with retention time indexing and migration times in capillary electrophoresis-mass spectrometry CE-MS (see Table 1 for a listing of the currently implemented functions). All these functions take base R data types as input and return base R data types facilitating portability and their re-use in other R packages. Exact masses for chemical formulas can be calculated for example with calculateMass(c(’C27H42O3’, ’C26H28O11’)), which returns the numeric c(414.3134, 516.1632) with the exact masses for the two compounds. Conversion between *m/z* values for ions and exact masses can be performed with the mass2mz() and mz2mass() functions, mass2mz(c(414.3134, 516.1632), ’[M+H]+’) would for example return the *m/z* values for the protonated ions of the provided compounds’ masses. Currently, 58 adducts from both positive and negative ionization mode are covered, but to maximize flexibility, the functions also support user provided adduct definitions.

The isotopologues() function in *MetaboCoreUtils* allows to identify and group mass peaks in a spectrum that potentially represents isotopologues of the same original compound based only on their *m/z* and intensity values, hence without any prior knowledge of chemical formulas or expected isotopes. This grouping is performed considering *m/z* differences induced by isotopic substitutions frequently observed in a reference database such as HMDB as well as substitution-specific observed ranges of intensity ratios between monoisotopic and isotopologue peaks. The function loops over the peaks in the spectrum, assumes the current peak being the monoisotopic peak of a certain compound and checks for peaks that are, in terms of *m/z* and intensity, compatible with one of the predefined most frequently observed substitutions, eventually grouping them into an isotopologue group.

Annotation-related functionality provided by the *MsCoreUtils* package comprises various spectra similarity scores (listed in Table 1) and the closest() function for matching of *m/z* values between spectra, accepting differences that can be expressed as an absolute error (tolerance) or an *m/z*-relative error (ppm). This function is used across all annotation functions (spectra similarity calculations but also MS^1^ annotations) and is implemented in C to ensure an optimal performance.

### 2.3. Creating, Managing and Using Reference Databases

Versioned and portable annotation resources are crucial for reproducible analyses. The *CompoundDb* package provides all functionality to create, manage and use portable SQLite-based databases providing compound annotations. The format of these databases (called CompDb) is flexible, hence supporting data import from many public resources including ChEBI [15], HMDB [16], MassBank [17] or PubChem [18]. If available, reference MS^2^ spectra can also be imported. In addition, through an object-oriented approach, the basic CompDb database format can be extended to the IonDb format and information about measured ions, such as adduct definition, *m/z*, retention time or MS^2^ spectra, can be added to an existing database. This enables users to combine and enrich publicly available annotations with the information from measurements of pure standards on specific LC–MS setups and thus to create reliable in-house reference databases. A related example is given in section “Annotation using reference *m/z* and retention times” of the Supplementary Material.

Importantly, these annotation databases are portable and can be versioned ensuring reproducibility. It is also planned to distribute pre-built CompDb annotation databases (e.g., for specific MassBank or HMDB releases) through Bioconductor’s *AnnotationHub*.

### 2.4. Functions for Working with Retention and Migration Times

Retention times represent a valuable orthogonal information to MS^1^ and MS^2^ for metabolite identification but are only partially transferable between laboratories. Even when using nominally the same separation chemistry, meaning the same column and eluents, differences in absolute retention times based on the used instrumentation can be observed. In GC-MS retention time indexing (RTI), e.g., based on a homologous series of *n*-alkanes, it has been suggested to normalize these effects [19]. Recently, a new retention indexing system for RP-LC-MS-based metabolomics was reported [20]. Further, the NORMAN network has demonstrated that RTI not only increases intra- and interlaboratory reproducibility, but can also be used as quality measure for different LC conditions [21]. Functions for such retention time indexing are also available in the *MetaboCoreUtils* package. The indexRtime() function is applicable to all of these systems. It accepts a numeric vector of retention times and a data.frame with the columns rtime and rindex as well as a function defining how the conversion from retention times to retention indices shall be performed. The default function uses linear interpolation but can be replaced by any other user-provided function. In addition, the function is independent of the substances used for indexing and can be generally adapted. An example illustrating this functionality is presented in the Supplementary Material.

Similar to retention times in GC- and LC-MS, migration times in CE-MS can vary between sample runs or even laboratories, sometimes even more strongly than retention times. Main variations in migrations times occur due to changes in the electroosmotic flow (EOF) between measurements, and these changes might appear for example due to adsorptions of analytes on the capillary wall or changes in the composition on the background electrolyte. Similar to retention time indexing, the migration time data can be normalized by converting them into an effective mobility, using mobility markers that were added to each sample. The effective mobility is responsible for separation of metabolites in CE and can be seen as a physicochemical property, which is stable in the same electrophoretic system (i.e., the same background electrolyte) and can be used to improve annotation of metabolites. It has already been shown that effective mobility transformation results in much more stable and reproducible results [22,23]. The *MetaboCoreUtils* package implements the convertMtime() function, which converts migration times into effective mobility values via the EOF markers used in the CE–MS run [24]. The function requires additional numerical vectors with the migration time of the markers, their corresponding effective mobility, the total capillary length, and the applied electrical field. Moreover, an optional numeric value with the time of the electrical field ramp (i.e., the time in which the electrical field ramps from 0 to 100%) can be provided. If two EOF markers are used, the function will not use information of the electrical field and capillary length, but it requires one marker to be the neutral marker, having an effective mobility of zero.

### 2.5. MS^1^ Annotation

Annotation of measured *m/z* values with potential metabolites, albeit representing the lowest level of annotation possible, is typically one of the first steps in metabolite identification workflows, and different tools for this task have been suggested [25,26]. MS^1^-based annotation involves matching of measured *m/z* values and/or retention times of LC–MS features against reference values. *MetaboAnnotation* provides the matchMz() function to perform such annotation in a user-friendly manner. This function supports a variety of input formats and different algorithms that can be chosen and configured using dedicated parameter objects (see Table 2). A simple *m/z* value-based annotation could be performed with matchMz(fts, db, Mass2MzParam(adduct = ’[M+H]+’, ppm = 10)) with fts being a data.frame with a column “mz” containing the *m/z* values for LC-MS features and db a CompDb database or a data.frame with a column “exactmass” that contains the exact masses of target compounds. Based on the user’s adduct definition, target *m/z* values will be calculated from these masses and matched against the query *m/z* values. Such annotation can also be performed on an *xcms* result with matchMz(featureDefinitions(xdata), db, Mass2MzParam(adduct = ’[M+H]+’, ppm = 10)) where xdata represents the *xcms* result object. More examples, including one for MS^1^-based annotation of an MZmine result, are provided in the Supplementary Material. The matching between query features and target compounds can be one-to-many or also one-to-none. The result object returned by the matchMz() function (called Matched) is specifically designed to simplify the handling of such rather complicated relationships to the user. It bundles the query and target object and internally represents the relationships between them in a data frame that also contains the score for each match. Filtering, sub setting, re-ordering and extracting matches, all potentially error prone operations, are all handled by the result object ensuring that the relationship between the query feature and target compound is preserved. In addition, the parameter object is stored along with all the input data within the result object, thus providing clear documentation on what settings the annotation result is based on.

### 2.6. MS^2^ Annotation

**Figure 2:**
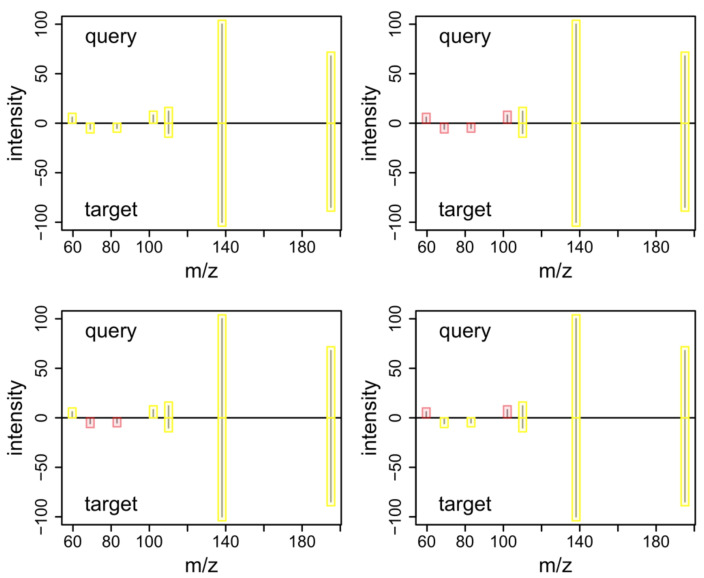
Peak mapping strategies of the joinPeaks() function. Peaks returned by the joining strategy are highlighted in yellow, those not considered in red. Mapping strategies are named according to the join terminology in SQL. Top left: the outer join option reports all peaks from both spectra. Top right: the inner join reports only matching peaks from both spectra. Bottom left: the left join option includes all peaks from the query spectrum and matching peaks from the target spectrum. Bottom right: the right join includes all peaks from the target spectrum and only matching peaks from the query spectrum.

The next step in metabolite identification after MS^1^ annotation involves the comparison of potentially generated experimental MS^2^ spectra against reference spectra, either from in-house or external libraries. The basic infrastructure for spectra similarity calculations is provided by the *Spectra* package with the compareSpectra() function. Spectra similarity calculations are performed as a two-step approach: first, peaks from the query and target spectrum are mapped to each other based on their *m/z* values and a given error (absolute or in ppm), and then a similarity score is calculated based on these matched peaks. A complete pairwise spectra similarity calculation between all spectra in Spectra objects a and b can be performed for example with compareSpectra(a, b, MAPFUN = joinPeaks, FUN = ndotproduct). Parameters MAPFUN and FUN allow for specification of the peak mapping function and the similarity score function, respectively. These can be one of the peak mapping functions listed in Table 3 (see also Figure 2 for peak mapping strategies) or similarity scores listed in Table 1. In addition, any of the similarity and distance quantification algorithms from the *philentropy* package [27] are supported, and even custom, user-provided, functions can be submitted. As a result, a matrix of pairwise similarity scores is returned.

**Figure 3:**
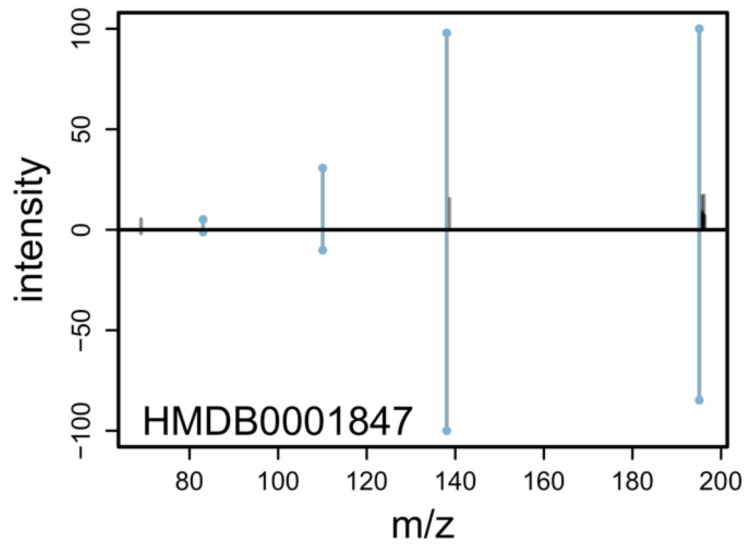
Mirror plot created with plotSpectraMirror for visual inspection of MS^2^ annotation results. The upper panel shows an experimental MS^2^ spectrum and the lower a reference spectrum for Caffeine from HMDB. Matching peaks are highlighted in blue.

The matchSpectra() function from the *MetaboAnnotation* package makes this powerful spectrum similarity calculation framework available to the end user, enabling an efficient and fast way to perform pairwise comparisons between experimental and reference MS^2^ spectra. Native parallelization is supported and can be configured with the *BiocParallel* package. Similar to the matchMz() function for MS^1^-based annotation, the algorithm can be configured with a dedicated parameter object (see Table 3), while the experimental query and reference target MS^2^ spectra have to be provided as Spectra objects. As an example, matchSpectra(qry, db, CompareSpectraParam()) would identify matching spectra between qry and db with a similarity score > 0.7 (default setting). The *Spectra* package natively supports import of MS data from mzML, mzXML and netCDF files and through its add-on packages *MsBackendMgf*, *MsBackendMsp* and *MsBackendMassbank* from files in MGF, MSP or MassBank format [17]. It is thus possible to use for example results from MS-DIAL or MZmine as input for matchSpectra by importing them with qry <- Spectra(’mzmine_result.mgf’, source = MsBackendMgf()). Alternatively, query MS^2^ spectra can also be directly extracted from a *xcms* result objects with qry <- featureSpectra(xdata, return.type = ’Spectra’). As target parameter (variable db in the example above), either a CompDb database or a Spectra object with data imported from MSP or MGF files can be used. Through the *MsBackendMassbank* package, it is also possible to directly interact with a MassBank SQL database hence not requiring import or export of thousands of individual records. The result returned from the matchSpectra() function (called MatchedSpectra) contains, similar to the result from the MS^1^-based annotation, the query and target data, the matching results, and scores as well as the parameter object. In addition, this object is designed to simplify the handling of the possible many-to-many matching results to the user. It provides functions to filter, subset, plot, and extract data. A visual inspection of two matched spectra could for example be performed with plotSpectraMirror(mtch[1]) where mtch is the result returned by a matchSpectra() call. This will create a mirror plot as shown in Figure 3. An example for MS^2^-based annotation is also provided in the Supplementary Material.

### 2.7. Examples and Use Cases

Examples and use cases are shown in the rendered R vignette provided as Supplementary Material, with the raw code and data files available in the GitHub repository https://github.com/jorainer/MetaboAnnotationTutorials (accessed on 4 February 2022). All required R packages as well as the raw data and vignette can be installed in R with BiocManager::install(’jorainer/MetaboAnnotationTutorials’) after first installing the *BiocManager* package with install.packages(’BiocManager’). In the Supplementary Material, the *MetaboAnnotation* package is used to MS^1^ annotate chromatographic peaks from a small LC-MS/MS dataset based only on *m/z* values as well as using *m/z* and retention times previously determined for a set of pure standards on the same LC–MS setup and instrumentation. Additional MS^2^-based annotation of the chromatographic peaks is performed by comparing experimental MS^2^ spectra against reference spectra from the Human Metabolome Database [32]. The reference annotation database for this example was created with the *CompoundDb* package based on HMDB 5.0 data release 2 November 2021 and contains annotations for 217,776 compounds as well as 64,920 MS^2^ spectra (predicted spectra were excluded).

Retention time indexing can help to make retention information more comparable. The example in section “Annotation using reference m/z and retention indices” of the supplement illustrates how functions from the *MetaboCoreUtils* and *MetaboAnnotation* packages can be combined to perform retention time indexing and annotation on the MS¹ level using *m/z* and retention index (RI). Picked and isotope grouped features from *C. elegans* metabolite extracts are subjected to retention time indexing, and annotation of metabolites is performed based on *m/z* and retention index instead of retention time.

Finally, examples for import and usage of analysis results from MZmine, together with the presented packages, are shown based on test files and results from [33].

Additional documentation is provided in the individual packages’ vignettes as well as at https://jorainer.github.io/SpectraTutorials/ (accessed on 4 February 2022).

## 3. Discussion

We implemented a set of R packages that together provides a comprehensive and modular infrastructure for untargeted metabolomics data annotation. While there exist already a considerable number of software packages with similar methodology, our packages provide in addition a rich infrastructure with functionalities ranging from reference implementations of established algorithms (some not available in R before) to routines for data import and export for a vast number of file formats as well as functions to efficiently handle, process, and filter MS data. Especially, the former helps against a frequently observed issue in R software development, which is the re-implementation of standard operations. Our packages export, next to the high-level functions, all their core functions that work with base R data types, hence enabling their re-use in other software. This will ultimately help to advance the field by allowing future developers to focus more on the implementation of improved annotation algorithms without the need to re-implement established methods or functions for MS data handling, import and export.

Another important aspect is the validity and correctness of algorithms and software. In contrast to many other R packages, we use comprehensive unit tests for all our functions (unit test coverage is above 90% for all our packages) that, if available, compare results with the results from the original reference implementations. These unit tests are evaluated daily by the Bioconductor build servers for all three main operating systems.

In addition, all our functions are well documented, and examples as well as comprehensive tutorials are provided. Unfortunately, many other R packages, especially those hosted only on GitHub, are only poorly documented, making their use difficult.

Lack of unit tests or poor documentation in original implementations were also reasons why some of the utility functions, albeit already available in other R packages such as *nontarget* or *Rdisop*, were re-implemented in our *MsCoreUtils* and *MetaboCoreUtils* packages. In addition, ultimately, we aim at collecting such frequently used core functionality in a single place, ideally through contributions from the original authors. The culture of open-first development and supportive attitude toward contributions is an incentive to support the ecosystem, instead of rewriting yet another implementation.

Next to documentation and unit tests, long-term support is also critical for the usefulness of software. There is a growing number of R packages for metabolomics data analysis being developed, but many of these are no longer maintained or updated after their initial publication or after the developing student moves on to a different job. By distributing the development on a large team of developers with different levels of seniority and from different institutions, we try to ensure long-term support of our code base. In addition, requiring code reviews from at least one other experienced developer adds to the robustness of the code and guarantees continuity even if individual developers should leave the team.

## 4. Materials and Methods

### 4.1. Acquisition of the Example Dataset for MS¹- and MS²-Based Annotation

Individual stock solutions of 15 analytical standards from different chemical classes were prepared by adding 1 mg of each of the following analytical standard into 1 mL of water (or other solvent, if specified): 1-methyluric acid (water + 5 µL sodium hydroxide 5 mM), 3-methylhistidine, ADMA, caffeine, CDP-choline, creatinine, dAMP, glutaric acid, glycero-phosphocholine, methionine, phenylpyruvic acid (ethanol), serine, sphingosine (methanol), taurine and threonic acid. Next, 200 µL of each standard solution were pipetted into a 5 mL Eppendorf tube, together with 40 µL of formic acid and 960 µL of ACN, resulting in 4 mL of a standard mix solution at 50 ppm. For data acquisition, 5 µL of standard mix solution was injected into a LC-MS system equipped with an Agilent 1290 UHPLC device (Agilent Technologies, Santa Clara, CA, USA) coupled with a SCIEX 5600 QTOF (AB Sciex LLC, Framingham, MA, USA). An Acquity UPLC BEH Amide Column (130 Å, 1.7 µm, 2.1 mm × 100 mm) was used in association with the respective pre-column. An IDA experiment (information dependent analysis, also known as data dependent analysis or DDA) was performed using a 50–1000 *m/z* range, with a 250 ms accumulation time for MS^1^ data. MS^2^ data were acquired using the same *m/z* range and a 1000 cps threshold. In addition, 50 mDa was used as mass tolerance with a maximum number of candidate ions per cycle set at 20. Dynamic background subtract and dynamic accumulation were also employed, with an accumulation time of 100 ms and collision energy set at 20 V.

### 4.2. Retention Indexing Example

For the retention indexing use case, MS^1^ data from [20] were used. Briefly, *Caenorhabditis elegans* N2 were extracted with 50% MeOH, and the extract was measured on a Supelco Ascentis Express C18 (150 × 2.1 mm, 3.0 µm particle size) using a Waters Acquity UPLC (Waters, Milford, MA, USA) coupled to a Bruker maXis plus UHR-ToF-MS (Bruker Daltonics, Bremen, Germany). Detailed experimental conditions as well as the retention index databases can be found in the original publication [20].

## 5. Conclusions

Here, we present the R packages *MetaboCoreUtils* and *MetaboAnnotation* that are well integrated with the R packages *Spectra*, *MsCoreUtils*, *MsBackendMgf*, *MsBackendMsp* and *MsBackendMassbank*, and thus together provide a modular and expandable ecosystem for the annotation of features from untargeted LC-MS and LC-MS/MS experiments using standard and established algorithms. In contrast to other, monolithic, annotation tools, our packages allow users to combine functions in a modular fashion. The ecosystem is expandable because external or user-defined functions can be used as alternatives to package-internal ones in the various annotation functions, e.g., to match peaks between compared spectra or to calculate similarities between these. The *CompoundDb* package provides, in addition, functionality to create, manage, and use reference databases that can be integrated directly into the annotation process. The infrastructure represented by our packages is thus a rich toolbox that enables building customized and reproducible annotation workflows in R. Even more importantly, all core functionality is exported, which enables its re-use in other software tools. Future developers of new, improved, annotation strategies can thus build upon our infrastructure without the need to implement again standard functionality for spectra comparison or functions to manipulate and handle MS data.

Finally, any functionality added to any of these packages requires code review from at least one other experienced core package developer from the RforMassSpectrometry initiative, ensuring high quality and reliability of the codebase.

The ecosystem will be further expanded in the future, for example, with functionality to group LC–ESI–MS features potentially representing ions from the same original compound (currently being implemented in the *xcms* and *MsFeatures* R packages) or the integration of yet other (public) reference libraries, databases and file formats (e.g., Agilent.cef or Bruker TimsTOF). Contributions from the community are highly welcome.

## Figures and Tables

**Table 1:** Listing of core utility functions for metabolite annotation. The first panel contains functions to work with chemical formulas followed by a panel with various utility functions. The last panel contains functions to calculate established spectra similarity scores.

Function	Description	Package
countElements	Counts elements in chemical formulas.	*MetaboCoreUtils*
pasteElements	Converts element counts to chemical formulas.	*MetaboCoreUtils*
subtractElements	Removes elements from chemical formulas.	*MetaboCoreUtils*
addElements	Adds elements to chemical formulas.	*MetaboCoreUtils*
standardizeFormula	Standardizes formulas according to the Hill notation [12].	*MetaboCoreUtils*
calculateMass	Calculates exact masses from chemical formulas.	*MetaboCoreUtils*
mass2mz, mz2mass	Converts between masses and *m/z* values.	*MetaboCoreUtils*
isotopologues	Groups potential isotopologue peaks in MS^1^ data.	*MetaboCoreUtils*
closest	Matches numeric values accepting differences.	*MsCoreUtils*
ndotproduct	Normalized dot product [13].	*MsCoreUtils*
neuclidian	Normalized Euclidian distance [13].	*MsCoreUtils*
navdist	Normalized absolute values distance [13].	*MsCoreUtils*
nspectraangle	Normalized spectra angle [14].	*MsCoreUtils*

**Table 2:** High-level functions to perform MS^1^ annotation. The algorithm used by matchMz can be selected and configured with a parameter object. Supported input objects are at present numeric, data.frame, SummarizedExperiment, CompDb and IonDb.

Function	Parameter Object	Description
matchMz	MzParam	Performs *m/z* matching between query and target.
matchMz	MzRtParam	Matches *m/z* values and retention times from query and target.
matchMz	Mass2MzParam	Performs *m/z* matching after converting target masses to *m/z* values.
matchMz	Mass2MzRtParam	Matches *m/z* values and retention times between query and target after conversion of target masses to *m/z* values.

**Table 3:** Functions for MS^2^-based annotation. The first panel contains function to map peaks between compared spectra. The second panel high-level functions to perform spectra similarity calculations.

Function	Parameter Object	Description
joinPeaks	-	Maps peaks between two spectra accepting differences between the peaks’ *m/z* values that can be defined by ppm and tolerance.
joinPeaksGnps	-	Hybrid search approach [28,29,30,31]: also peaks for which the difference in *m/z* values matches the difference of the precursor *m/z* of the two spectra are considered matching.
compareSpectra	-	Calculates pairwise similarity scores between two spectra objects.
matchSpectra	CompareSpectraParam	Identifies spectra with a similarity score above a user-defined threshold.
matchSpectra	MatchForwardReverseParam	Identifies spectra with a similarity score above a user-defined threshold and calculates in addition the reverse score.

## Data Availability

The data presented in this study are openly available in the GitHub repository https://github.com/jorainer/MetaboAnnotationTutorials (accessed on 4 February 2022).

## Supplementary Materials

The following supporting information can be downloaded at: https://www.mdpi.com/article/10.3390/metabo12020173/s1, Supplementary File S1: pdf document with use cases and examples.
