# Supplementary material for: A Modular and Expandable Ecosystem for Metabolomics Data Annotation in R
Source: Metabolites. 2022 Feb 11;12(2):173. doi: 10.3390/metabo12020173 (PMC8878271; doi:10.3390/metabo12020173)
Supplement: Supplementary file 1 [file metabolites-12-00173-s001.zip › metabolites-1573991-supplementary.pdf]

Supplementary document for: *A modular and expandable ecosystem for metabolomics data annotation in R*

Johannes Rainer, Andrea Vicini, Liesa Salzer, Jan Stanstrup, Josep M Badia, Steffen Neumann, Michael Stravs, Vinicius Verri Hernandes, Laurent Gatto, Sebastian Gibb and Michael Witting

This documents provides use cases describing the annotation of untarget metabolomics and small compound datasets using functionality from the *MetaboAnnotation*, *MetaboCoreUtils* and *CompoundDb* packages. All data used in this document is available within the github repository https://github.com/jorainer/MetaboAnnotationTutorials. Additional use cases and examples can be found in the packages' vignettes and workshops/tutorials. See section *Package repositories and additional tutorials* for links to the package repositories and tutorials.

Installation instructions

The R packages as well as the example files used in this document can be installed with the R code below:

```
install.packages("BiocManager")
BiocManager::install("jorainer/MetaboAnnotationTutorials")
```

The source code for this document along with the test data can be downloaded from the github repository https://github.com/jorainer/MetaboAnnotationTutorials with the command (or alternatively downloading the zip archive directly from the github page).

```
git clone https://github.com/jorainer/MetaboAnnotationTutorials
```

MS^1 annotation

In this example we annotate chromatographic peaks identified in a test sample provided within this repository first based only on their *m/z* values and then using their *m/z* and retention times against previously determined values for a set of pure standards. The test data represents LC-MS/MS measurements of a set of metabolite standards. In brief, aliquots for pure standards of 15 metabolites were solved in water and injected into a LC-MS system. Details are provided in the Material and Methods section in the main text.

Below we load the data which is distributed within the `MetaboAnnotationTutorials` package and perform the chromatographic peak detection using the *xcms* package.

```
library(xcms)

fl <- system.file("mzML", "HighIS_Mix07_CE20_POS.mzML",
                  package = "MetaboAnnotationTutorials")

std_data <- readMSData(fl, mode = "onDisk")
```

We next perform the chromatographic peak detection using the *centWave* algorithm followed by a *peak refinement* to reduce the number of peak detection artifacts, such as split-peaks or overlapping peaks (see the *LC-MS/MS data analysis with xcms* vignette from the `xcms` package for details on the chromatographic peak detection settings).

```
#' Peak detection
cwp <- CentWaveParam(snthresh = 10, prefilter = c(3, 4000), ppm = 40,
                     peakwidth = c(2, 8), integrate = 2)
std_data <- findChromPeaks(std_data, param = cwp)
#' Peak refinement
std_data <- refineChromPeaks(
    std_data, MergeNeighboringPeaksParam(expandRt = 3))
```

In total 175 chromatographic peaks have been identified. This is even more than would be expected since the sample represents a mixture of 15 pure standards solved in water. Below we display the first 6 of the detected chromatographic peaks.

```
head(chromPeaks(std_data))
```

```
##              mz     mzmin     mzmax     rt  rtmin  rtmax       into       intb
## CP001 355.28240 355.28193 355.28369 30.495 24.575 37.641  83909.909  78939.997
## CP002 358.98005 358.97954 358.98037 10.390 10.065 11.356   7002.894   6897.381
## CP003 413.26607 413.26573 413.26704 32.892 25.729 37.641 102217.289 100770.550
## CP004 301.14091 301.13962 301.14279 25.729 24.575 40.013  47095.520  44283.920
## CP005  80.02494  80.02419  80.02543 40.013 35.261 56.554  53223.003  53005.070
## CP006  63.99953  63.99950  63.99958 42.371 35.261 61.268 156775.850 156184.543
##            maxo  sn sample
## CP001 10329.126  25      1
## CP002  5138.685  32      1
## CP003 22355.266  90      1
## CP004  7085.818  40      1
## CP005  6082.308 181      1
## CP006 17705.469 242      1
```

We next annotate these chromatographic peaks using only their m/z values. As a reference we load a `CompDb` database containing all compounds from the Human Metabolom Database (Wishart et al. 2021) version 5.0 which is also provided as a data release on the `MetaboAnnotationTutorials` github repository. The database was build from HMDB 5.0 (2021-11-02) using the *CompoundDb* package (see the package documentation for more information).

Below we download the database from github to a temporary folder.

```
#' Download the CompDb database using curl
library(curl)
dbname <- "CompDb.Hsapiens.HMDB.5.0.sqlite"
db_file <- file.path(tempdir(), dbname)
curl_download(
    paste0("https://github.com/jorainer/MetaboAnnotationTutorials/",
           "releases/download/2021-11-02/", dbname),
    destfile = db_file)
```

We next load the database using the `CompoundDb` package.

```r
library(CompoundDb)
#' Load a CompDb database with compound annotation from HMDB
cdb <- CompDb(db_file)
cdb
```

```
## class: CompDb
##  data source: HMDB
##  version: 5.0
##  organism: Hsapiens
##  compound count: 217776
##  MS/MS spectra count: 64920
```

Note: while we are here annotating chromatographic peaks from an `xcms` result object, it would also be possible to annotate LC-MS features using the same approach by simply exchanging the `chromPeaks` call with `featureDefinitions`. In addition, the annotation could also be performed on a `SummarizedExperiment` representing the preprocessing results which can be generated from an `xcms` result object using the `quantify` method.

Annotation using m/z values

We next extract all peaks from the `xcms` result object and annotate them using the `matchMz` function against all compounds from HMDB (the compound annotations can be extracted from the `CompDb` database with the `compounds` function). The reference database does not provide m/z values but only exact masses for each compound. Thus, in order to annotate our chromatographic peaks' m/z values, m/z values for expected ions of the compounds need to be calculated first. With `Mass2MzParam` we can configure the parameters for the annotation. Parameter `adducts` allows to specify the adducts (ions) we expect to see mostly in the data set. For our example we assume to see mostly `[M+H]+` and `[M+Na]+` positive ions. With parameters `tolerance` and `ppm` we can finally define also the absolute and/or m/z relative acceptable difference, respectively, for m/z values to be considered matching.

```r
library(MetaboAnnotation)

#' Get the chromatographic peaks and add a column with the peak_id
pks <- data.frame(peak_id = rownames(chromPeaks(std_data)),
                  chromPeaks(std_data))

#' Set parameters for the m/z-based annotation
param <- Mass2MzParam(adducts = c("[M+H]+", "[M+Na]+"),
                      tolerance = 0, ppm = 10)

#' Perform the matching
pks_match <- matchMz(
    pks,
    compounds(cdb, c("compound_id", "exactmass", "formula", "name")),
    param = param)

pks_match
```

```
## Object of class Matched
## Total number of matches: 697
## Number of query objects: 175 (112 matched)
## Number of target objects: 217776 (393 matched)
```

The result object handles the potential many-to-many matching between chromatographic peaks (*query*) and reference (*target*) and contains all information from both the query and the target object along with the *score* for the match (in this case the difference between the *m/z* values). Below we list all available information in the result object.

```
colnames(pks_match)
```

```
##  [1] "peak_id"            "mz"                 "mzmin"
##  [4] "mzmax"              "rt"                 "rtmin"
##  [7] "rtmax"              "into"               "intb"
## [10] "maxo"               "sn"                 "sample"
## [13] "target_compound_id" "target_name"        "target_formula"
## [16] "target_exactmass"   "adduct"             "score"
```

The n:m mapping is represented in the `matches` data frame. It maps each annotated query element to all matching target elements. Below we list the first 6 rows from that table. Column `"score"` provides the difference between the query and target *m/z* values (in Da).

```
head(matches(pks_match))
```

```
##   query_idx target_idx adduct       score
## 1         1       6977 [M+H]+ 0.001888540
## 2         1       7006 [M+H]+ 0.001888540
## 3         1     158233 [M+H]+ 0.001888540
## 4         1     160274 [M+H]+ 0.001888540
## 5         1     170679 [M+H]+ 0.001888540
## 6         2     164617 [M+H]+ 0.003242238
```

For users it's however more convenient to extract annotation information with the `matchedData` function, that will return a data frame with all annotations. If query elements are annotated to more than one target element they will be listed in as many rows as there are matches. Not matching query elements are represented with a single row in the result table, containing `NA` for all annotations. Below we extract all annotations, restricting to some of the annotation columns.

```
matchedData(pks_match, c("peak_id", "target_name",
                         "target_formula", "adduct"))
```

```
## DataFrame with 760 rows and 4 columns
##               peak_id            target_name target_formula      adduct
##           <character>            <character>    <character> <character>
## CP001           CP001 MG(0:0/18:2(9Z,12Z)/..       C21H38O4      [M+H]+
## CP001.1         CP001 MG(18:2(9Z,12Z)/0:0/..       C21H38O4      [M+H]+
## ...               ...                    ...            ...         ...
## CPM188         CPM188                     NA             NA          NA
## CPM189         CPM189                     NA             NA          NA
```

As we can see, the number of rows of this data frame is much larger than the number of chromatographic peaks. Most peaks are in fact annotated to several possible compounds based on their *m/z*. The average number of compounds to which a chromatographic peak in this data is annotated is calculated below.

```
matches(pks_match)[, 1] |>
table() |>
mean()
```

```
## [1] 6.223214
```

Annotations in the result object are organized by query, annotations for individual query elements can thus be accessed very easily. Below we list all annotations for the first chromatographic peak.

```
matchedData(pks_match[1], c("peak_id", "target_name",
                            "target_formula", "adduct"))
```

```
## DataFrame with 5 rows and 4 columns
##              peak_id            target_name target_formula      adduct
##          <character>            <character>    <character> <character>
## CP001          CP001 MG(0:0/18:2(9Z,12Z)/..       C21H38O4      [M+H]+
## CP001.1        CP001 MG(18:2(9Z,12Z)/0:0/..       C21H38O4      [M+H]+
## CP001.2        CP001 Glyceryl monolinoleate       C21H38O4      [M+H]+
## CP001.3        CP001   2-Linoleoyl Glycerol       C21H38O4      [M+H]+
## CP001.4        CP001             RIOPROSTIL       C21H38O4      [M+H]+
```

All compounds have thus the exact same chemical formula and hence, using only the m/z value, we can not discriminate between them or determine which would be the correct annotation. Considering also the retention times would allow a better annotation and increase its confidence.

Annotation using reference m/z and retention times

For LC-MS experiments compounds are first chromatographically separated based on some property of the compound (other than their mass). Thus, also the retention times contain information that can improve the annotation. In this section we use information from a previous experiment in which pure standards were measured with the same LC-MS setup used to generate the present test data and in which the retention time as well as the ion (adduct) and its m/z value were determined. These reference values were thus empirically determined on the same instruments with the same setup and should thus help to get higher confidence annotations.

Below we load the reference retention time and m/z data for some of the measured standards.

```
fl <- system.file("txt", "std_ions.txt",
                  package = "MetaboAnnotationTutorials")
std_ions <- read.table(fl, sep = "\t", header = TRUE)
head(std_ions)
```

```
##   compound_id ion_adduct ion_rt   ion_mz
## 1 HMDB0001539     [M+H]+    176 203.1503
## 2 HMDB0000905     [M+H]+    208 332.0754
## 3 HMDB0001847     [M+H]+     35 195.0877
## 4 HMDB0001413     [M+H]+    217 490.1224
## 5 HMDB0000562     [M+H]+     87 114.0662
## 6 HMDB0000086     [M+H]+    189 258.1101
```

We can now annotate the chromatographic peaks using this reference data. We configure the matching using the `MzRtParam`, which allows to annotate query elements based on *m/z* and retention times. With `ppm` we define again the *m/z*-relative acceptable difference of the *m/z* values and with `toleranceRt` the absolute acceptable difference in measured retention times. The parameters `mzColname` and `rtColname` allow us to define the names of the columns in the query and target objects containing the *m/z* values and retention time values for the matching. Settings for `ppm` and `toleranceRt` should be adapted to the experimental setup, retention time difference could e.g. be larger for HILIC-based chromatographic separation. Below we use a difference of 7 seconds (since retention times in `std_ions` and in the `xcms` result are reported in seconds).

```
param <- MzRtParam(ppm = 10, toleranceRt = 7)
pks_match <- matchMz(pks, std_ions, param = param,
                     mzColname = c("mz", "ion_mz"),
                     rtColname = c("rt", "ion_rt"))
pks_match
```

```
## Object of class Matched
## Total number of matches: 9
## Number of query objects: 175 (9 matched)
## Number of target objects: 19 (9 matched)
```

In addition to supplying the reference (target) data as a `data.frame` we could also directly use an `IonDb`. This type of annotation object extends the above used `CompDb` database providing in addition to general compound annotations also information on measured ions including their retention times and *m/z* values. It is thus designed to contain reference annotation databases for specific LC-MS setups and MS instrumentation used in a lab. Below we create such an `IonDb` database (which will be saved in the present example to a temporary file) from the HMDB `CompDb` database and add our ion annotations to it.

```
#' Create an IonDb filling it with data from cdb.
idb <- IonDb(tempfile(), cdb)

#' Insert measured m/z and retention times for ions
idb <- insertIon(idb, std_ions)
idb
```

```
## class: IonDb
##  data source: HMDB
##  version: 5.0
##  organism: Hsapiens
##  compound count: 217776
##  MS/MS spectra count: 64920
##  ion count: 19
```

The advantage of such an annotation database over individual (text) file-based annotation approaches is obvious: along with the annotation data also metadata can be saved and the database can be versioned, which is crucial for reproducible workflows. Also, the database's SQLite file is portable and can be distributed and used on different computers. In addition, it would also be possible to use a MySQL-based database system which would enable centralized annotation resources in a laboratory. More information on the creation and usage of annotation resources with the `CompoundDb` package is provided in its package vignettes which are also available here.

We can now annotate our peaks against that database using the same call as above, but providing the `idb` as `target` parameter.

```r
pks_match <- matchMz(
    pks, ions(idb, c("compound_id", "ion_adduct", "ion_mz", "ion_rt", "name")),
    param = param, mzColname = c("mz", "ion_mz"),
    rtColname = c("rt", "ion_rt"))
pks_match
```

```
## Object of class Matched
## Total number of matches: 9
## Number of query objects: 175 (9 matched)
## Number of target objects: 19 (9 matched)
```

In total 9 chromatographic peaks were annotated to an ion of one of the standards. We can identify the chromatographic peaks with available annotation using the `whichQuery` function, that returns their index in the query. Below we thus also subset the matched results to only those chromatographics peaks with an annotation.

```r
whichQuery(pks_match)
```

```
## [1]  61  68  74  86  88 131 142 148 162
```

```r
pks_match <- pks_match[whichQuery(pks_match)]
```

We next build a result table for the successfully annotated chromatographic peaks.

```r
library(pander)
pandoc.table(as.data.frame(
    matchedData(pks_match, c("peak_id", "target_name", "score", "score_rt"))),
    style = "rmarkdown", split.tables = Inf)
```

	peak_id	target_name	score	score_rt
**CP067**	CP067	Caffeine	0.0006847	0.261
**CP074**	CP074	Creatinine	0.0001926	0.238
**CP080**	CP080	Sphingosine	0.0003391	3.013
**CP094**	CP094	L-Methionine	0.0001487	6.839
**CP096**	CP096	1-Methyluric acid	0.0002982	0.626
**CP143**	CP143	3-Methylhistidine	0.0002466	0.734
**CP154**	CP154	Asymmetric dimethylarginine	0.0003642	3.688
**CP162**	CP162	Glycerophosphocholine	0.0003917	0.098
**CP176**	CP176	Deoxyadenosine monophosphate	0.0007856	2.337

Columns `"score"` and `"score_rt"` contain the difference between the query and the annotated target's m/z (in Da) and retention time (in seconds), respectively.

Annotation using reference m/z and retention indices

Retention time is a valuable orthogonal information for metabolite identification, but chromatographic conditions are far from being standardized. Even when using nominally the same chromatographic system, meaning column and eluents, differences in retention times arise due to dead volumes, gradient delay volumes, etc. In GC-MS retention time indexing has been used to normalize for drifts in retention times by

converting them into retention indices. A similar approach has been recently described for LC-MS (Stoffel et al. 2021) using N-Alkyl-pyridinium sulfonates as retention indexing substances. Their use allows to convert retention times into retention indices and to compare retention information across different LC-MS systems.

We first load data from an untargeted metabolomic experiments. This data has been completely processed. Each feature is identified by a unique name.

```
library(MetaboCoreUtils)

fl <- system.file("txt", "rti_ms_pos_features.txt",
                  package = "MetaboAnnotationTutorials")
ms1_features <- read.table(fl, header = TRUE, sep = "\t")
head(ms1_features)
```

```
##            Name       mz    rtime Cel_pure_1.B.1_01_21144
## 1 Cluster_00003 71.01397 1.527515                946.2993
## 2 Cluster_00004 72.33667 1.529377               1402.1213
## 3 Cluster_00006 86.37420 1.946073               7245.5283
## 4 Cluster_00018 66.03701 1.454697               1306.3455
## 5 Cluster_00025 71.03044 1.259239             256197.7500
## 6 Cluster_00030 80.03526 1.259666             242820.3125
##   Cel_pure_1.B.1_01_21149 Cel_pure_1.B.1_01_21154 Cel_pure_1.B.1_01_21159
## 1                 806.088                 796.521                771.9626
## 2                1427.545                1334.492               1400.0985
## 3                8062.006                8196.895               8502.9092
## 4                1389.757                1338.029               1341.1783
## 5              238996.312              212299.125             242366.2188
## 6              209218.312              174476.219             223134.0000
##   Cel_pure_1.B.1_01_21164
## 1                 713.608
## 2                1503.299
## 3                9668.902
## 4                1584.487
## 5              239547.453
## 6              215851.828
```

Likewise we load the information on the N-Alkyl-pyridinium sulfonates. The retention index is defined as the number of carbons in the alkyl chain times 100.

```
fl <- system.file("txt", "rti_ms_pos_naps.txt",
                  package = "MetaboAnnotationTutorials")
ms1_naps <- read.table(fl, header = TRUE, sep = "\t")
head(ms1_naps)
```

```
##   rtime rindex
## 1    NA    100
## 2   1.5    200
## 3   1.7    300
## 4   2.6    400
## 5   5.9    500
## 6  10.3    600
```

The relationship between the retention time and retention index can be plotted.

```
plot(ms1_naps)
```

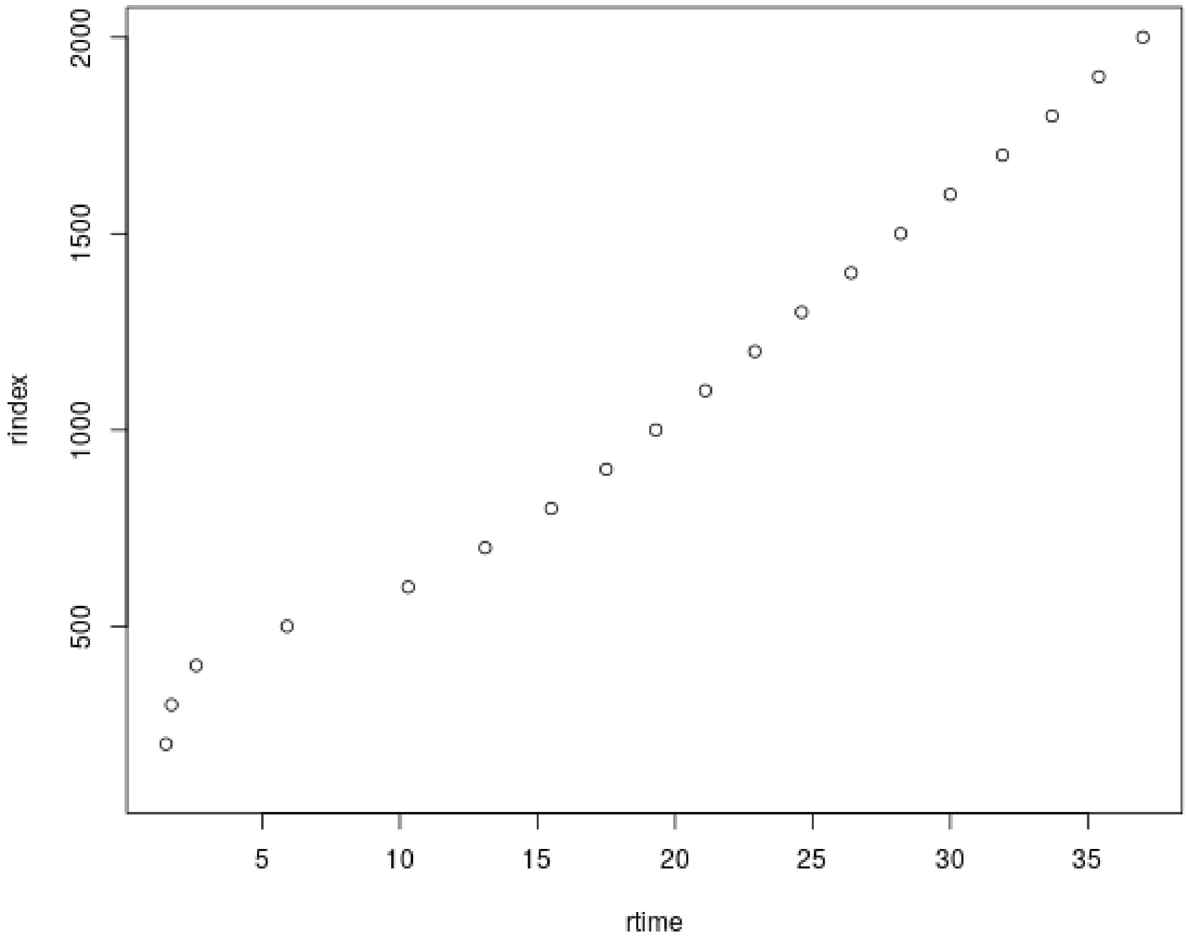

We can use the N-Alkly-pyridinium sulfonates to convert the retention times of the measured features using the `indexRtime` function. By default linear interpolation is used for conversion, but other (custom) functions can be defined if required.

```
ms1_features$rindex <- indexRtime(ms1_features$rtime, ms1_naps)
```

This data can then be annotated using reference databases. Two different databases are used. One was obtained on a UHPLC column and one on a HPLC column of the same column chemistry. The untargeted data was obtained from a HPLC column, but using a different LC hardware, resulting in different absolute retention times.

```
fl <- system.file("txt", "rti_db_hplc.txt",
                  package = "MetaboAnnotationTutorials")
db_hplc <- read.table(fl, header = TRUE, sep = "\t",
                      quote = "", comment.char = "")
```

```r
fl <- system.file("txt", "rti_db_uhplc.txt",
                  package = "MetaboAnnotationTutorials")
db_uhplc <- read.table(fl, header = TRUE, sep = "\t",
                       quote = "", comment.char = "")
```

Next we can define the `Mass2MzRtParam` similar to above. We will use an absolute error of 10 retention index units and use `[M+H]+`and `[M+Na]+` adducts.

```r
match_param <- Mass2MzRtParam(adducts = c("[M+H]+", "[M+Na]+"),
                              tolerance = 0.005,
                              toleranceRt = 10)
```

Matching can now be performed. Important is that the column with the retention index needs to be defined using `rtColname`. Matching is performed against both databases and results are then filtered to only contain features with at least one match.

```r
matches_hplc <- matchMz(ms1_features,
                        db_hplc,
                        match_param,
                        massColname = "exact.mass",
                        mzColname = "mz",
                        rtColname = "rindex")

matches_hplc <- matches_hplc[whichQuery(matches_hplc)]

matches_uhplc <- matchMz(ms1_features,
                         db_uhplc,
                         match_param,
                         massColname = "exact.mass",
                         mzColname = "mz",
                         rtColname = "rindex")

matches_uhplc <- matches_uhplc[whichQuery(matches_uhplc)]
```

We next build a result table for the successfully annotated chromatographic peaks. First for the results matching with the HPLC database.

```r
library(pander)
pandoc.table(head(as.data.frame(
  matchedData(matches_hplc, c("Name", "mz", "rindex", "target_name",
                              "adduct", "score", "score_rt")))),
  style = "rmarkdown", split.tables = Inf)
```

	Name	mz	rindex	target_name	adduct	score	score_rt
**24**	Cluster_00116	132.1	328.8	5-AMINOLEVULINATE	[M+H]+	0.005076	1.226
**24.1**	Cluster_00116	132.1	328.8	N-ACETYLALANINE	[M+H]+	0.005076	1.226
**395**	Cluster_01443	125.1	418.4	4-METHYLCATECHOL	[M+H]+	0.0002352	8.425
**434**	Cluster_01544	133	392.7	GLUTARATE	[M+H]+	0.000176	0.733
**434.1**	Cluster_01544	133	392.7	GLUTARATE	[M+H]+	0.000176	2.267
**469**	Cluster_01596	138.1	582.2	SALICYLAMIDE	[M+H]+	0.0001774	1.791

Then for matching with the UPLC database.

```
library(pander)
pandoc.table(head(as.data.frame(
  matchedData(matches_uhplc, c("Name", "mz", "rindex", "target_name",
                               "adduct", "score", "score_rt")))),
  style = "rmarkdown", split.tables = Inf)
```

	Name	mz	rindex	target_name	adduct	score	score_rt
**24**	Cluster_00116	132.1	328.8	N-ACETYLALANINE	[M+H]+	0.005076	4.774
**380**	Cluster_01397	123	679.6	BENZOATE	[M+H]+	0.0003409	1.38
**434**	Cluster_01544	133	392.7	GLUTARATE	[M+H]+	0.000176	4.733
**434.1**	Cluster_01544	133	392.7	GLUTARATE	[M+H]+	0.000176	3.733
**469**	Cluster_01596	138.1	582.2	4-AMINOBENZOATE	[M+H]+	0.0001774	2.791
**469.1**	Cluster_01596	138.1	582.2	ANTHRANILATE	[M+H]+	0.0001774	2.791

MS^2 annotation

In addition to MS^1 annotation described in the previous section, it is also possible to annotate features (or chromatographic peaks) by comparing experimentally measured MS^2 (MS/MS) spectra against reference spectra. Such annotation can be performed using the `matchSpectra` from the `MetaboAnnotation` package that takes `Spectra` objects (with MS^2 spectra) as query and target parameters. Such `Spectra` objects can be either imported from a variety of input formats, including MGF, MSP, mzML, mzXML or netCDF files, extracted from `xcms` result objects or even retrieved from databases such as *MassBank* (see also the tutorials from SpectraTutorials for more examples and use cases).

To illustrate this, we will extract in this section MS^2 spectra for the annotated chromatographic peaks from the previous section and compare them against reference MS^2 spectra from HMDB. Annotation reliability can be increased if, in addition to m/z and retention time, also the MS^2 spectra for a chromatographic peak or a feature matches the reference spectrum from a pure standard.

We first extract all MS^2 spectra for the annotated chromatographic peaks using the `chromPeakSpectra` function from the `xcms` result object. This will return all MS^2 spectra with the precursor m/z and retention time within the m/z ranges and retention time ranges of the detected chromatographic peaks. For an `xcms` result object containing also correspondence results (i.e. grouped chromatographic peaks across samples, also referred to as *features*) the `featureSpectra` function could be used instead.

```
std_spectra <- chromPeakSpectra(std_data, return.type = "Spectra",
                                peaks = pks_match$peak_id)
std_spectra
```

```
## MSn data (Spectra) with 11 spectra in a MsBackendMzR backend:
##           msLevel     rtime scanIndex
##         <integer> <numeric> <integer>
## F1.S107         2    35.650       107
## F1.S193         2    66.622       193
## ...           ...       ...       ...
## F1.S707         2   209.046       707
## F1.S760         2   221.347       760
##  ... 38 more variables/columns.
##
## file(s):
## HighIS_Mix07_CE20_POS.mzML
```

For some of the chromatographic peaks we have more than one MS^2 spectrum, but in total we have MS^2 spectra only for 7 chromatographic peaks. Below we count the number of spectra per peak.

```
table(std_spectra$peak_id)
```

```
##
## CP067 CP074 CP080 CP143 CP154 CP162 CP176
##     1     2     2     2     1     1     2
```

Before matching the spectra we are *cleaning* our experimental spectra removing peaks with an intensity lower than 5% of a spectra's highest peak intensity and removing spectra with less than 3 peaks.

```
#' Define a function to remove low intensity peaks
low_int <- function(x, ...) {
    x > max(x, na.rm = TRUE) * 0.05
}
#' Remove peaks with an intensity below 5% of BPI
std_spectra <- filterIntensity(std_spectra, intensity = low_int)

#' Remove peaks with less than 3 peaks
std_spectra <- std_spectra[lengths(std_spectra) > 2]
std_spectra
```

```
## MSn data (Spectra) with 8 spectra in a MsBackendMzR backend:
##           msLevel     rtime scanIndex
##         <integer> <numeric> <integer>
## F1.S107         2    35.650       107
## F1.S127         2    38.323       127
## ...           ...       ...       ...
## F1.S600         2   188.408       600
## F1.S707         2   209.046       707
##  ... 38 more variables/columns.
##
## file(s):
## HighIS_Mix07_CE20_POS.mzML
## Lazy evaluation queue: 1 processing step(s)
## Processing:
##  Remove peaks based on their intensities and a user-provided function in spectra of MS level(s) 2. [
```

In addition, we scale the peak intensities within each spectrum to values between 0 and 100. This is mostly for visualization reason, since most spectra similarity scoring algorithms are independent of absolute peak intensities.

```
#' Define a function to *scale* the intensities
scale_int <- function(x, ...) {
    maxint <- max(x[, "intensity"], na.rm = TRUE)
    x[, "intensity"] <- 100 * x[, "intensity"] / maxint
    x
}
#' *Apply* the function to the data
std_spectra <- addProcessing(std_spectra, scale_int)
```

Below we match now these *cleaned* spectra against the reference spectra from HMDB. We configure the matching using the `CompareSpectraParam` allowing m/z differences of 50ppm between peaks from a query and a target spectrum. Since HMDB does not provide precursor m/z we in addition have to disable the additional matching filter (i.e. set `requirePrecursor = FALSE`), which has however a negative impact on the performance of the comparisons. By default, spectra similarities (calculated with the normalized dot product) higher than 0.7 will be considered matching.

```
spectra_match <- matchSpectra(
    std_spectra, Spectra(cdb),
    param = CompareSpectraParam(ppm = 50, requirePrecursor = FALSE))
spectra_match
```

```
## Object of class MatchedSpectra
## Total number of matches: 27
## Number of query objects: 8 (4 matched)
## Number of target objects: 64920 (27 matched)
```

Thus, in total 4 of the query spectra were matched with 27. Below we create a result table for this matching.

```
res <- spectraData(spectra_match,
                   c("peak_id", "target_compound_id", "score"))
cmp_id <- unique(res$target_compound_id)
cmp_id <- cmp_id[!is.na(cmp_id)]
cmps <- compounds(cdb, filter = ~ compound_id == cmp_id, "name")
rownames(cmps) <- cmps$compound_id

res$name <- cmps[res$target_compound_id, "name"]
rownames(res) <- NULL
pandoc.table(as.data.frame(res),
    style = "rmarkdown", split.tables = Inf)
```

peak_id	target_compound_id	score	name
CP067	HMDB0001847	0.7258	Caffeine
CP067	HMDB0001847	0.705	Caffeine
CP067	HMDB0001847	0.7016	Caffeine
CP067	HMDB0001847	0.7046	Caffeine
CP080	NA	NA	NA
CP080	HMDB0253992	0.7589	Laurocapram
CP080	HMDB0253992	0.7789	Laurocapram
CP143	NA	NA	NA
CP143	HMDB0000479	0.8739	3-Methylhistidine
CP143	HMDB0000479	0.8739	3-Methylhistidine
CP154	NA	NA	NA
CP162	NA	NA	NA
CP176	HMDB0000034	0.7165	Adenine
CP176	HMDB0000034	0.7094	Adenine
CP176	HMDB0000034	0.7092	Adenine
CP176	HMDB0000034	0.7133	Adenine
CP176	HMDB0000034	0.7165	Adenine
CP176	HMDB0000034	0.7123	Adenine
CP176	HMDB0000034	0.7165	Adenine

peak_id	target_compound_id	score	name
CP176	HMDB0000034	0.7165	Adenine
CP176	HMDB0000034	0.7139	Adenine
CP176	HMDB0000050	0.7067	Adenosine
CP176	HMDB0000050	0.7165	Adenosine
CP176	HMDB0000101	0.7165	Deoxyadenosine
CP176	HMDB0000905	0.9523	Deoxyadenosine monophosphate
CP176	HMDB0000905	0.8143	Deoxyadenosine monophosphate
CP176	HMDB0000905	0.7407	Deoxyadenosine monophosphate
CP176	HMDB0000905	0.9136	Deoxyadenosine monophosphate
CP176	HMDB0000905	0.9523	Deoxyadenosine monophosphate
CP176	HMDB0000905	0.9524	Deoxyadenosine monophosphate
CP176	HMDB0001983	0.7086	5'-Deoxyadenosine

Matching results can also be very easily visualized with the `plotSpectraMirror` function. Below we show the results for the first peak (Caffeine). The upper spectrum represents the query, the lower the target (reference) spectrum.

```
plotSpectraMirror(spectra_match[1])
```

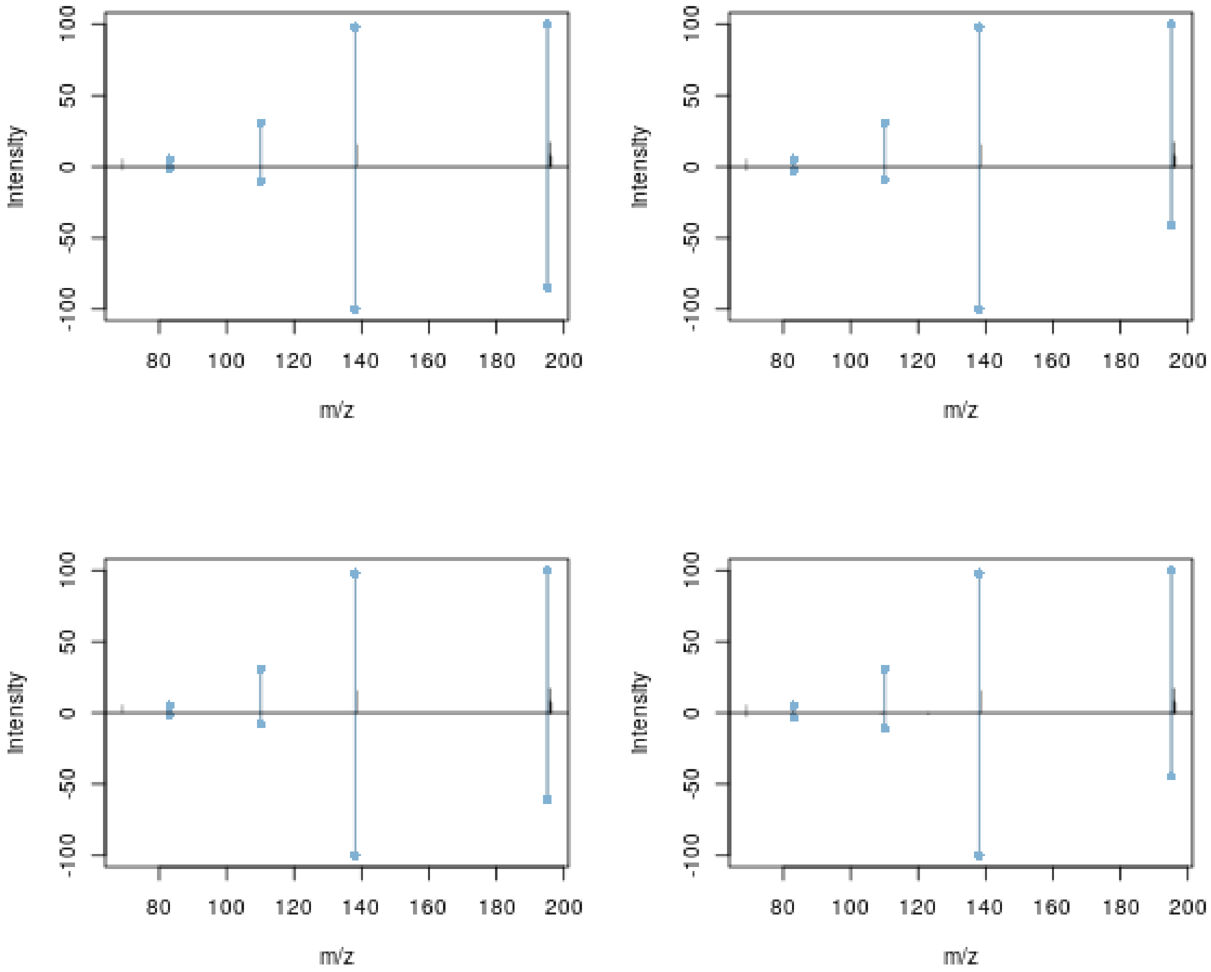

The results seem to be reasonable. For the second peak (third spectrum) the match was based however on a single peak.

```
plotSpectraMirror(spectra_match[3])
```

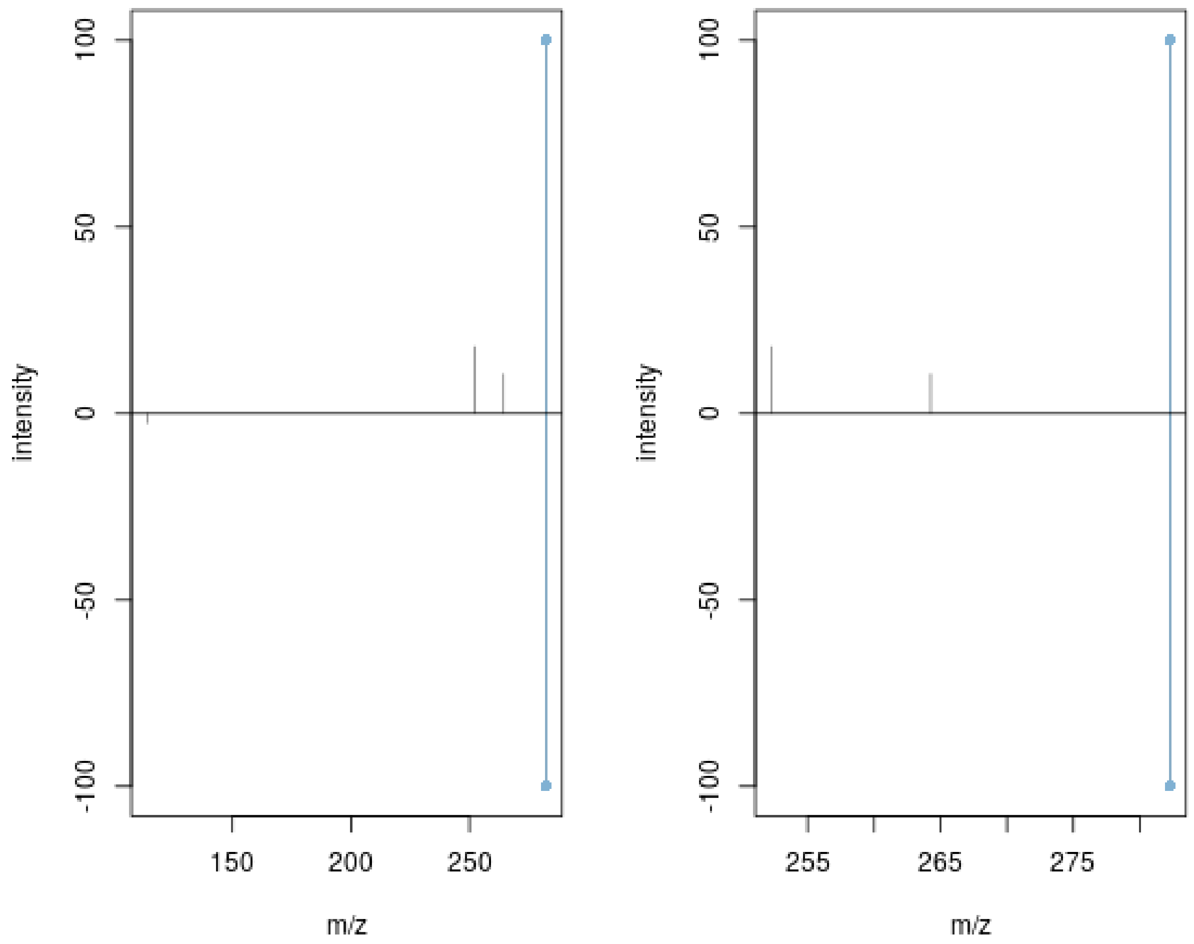

Note that we could now also add some of the (cleaned and annotated) MS^2 spectra from the present test set, to the `IonDb` using the `insertSpectra` function. This enables generation of lab-internal reference databases including also MS/MS spectra.

Working with data from other tools

MS^1-based annotations can be performed on any `data.frame` or similar that provides m/z and/or retention time values. For MS^2-based annotations a `Spectra` object would be needed, and that can be imported from a variety of formats. In this section we show how pre-processing results from an external tool (in this case MZmine) can be imported and used as input for `MetaboAnnotation`.

The test data we are using is one of the example data sets for GNPS Feature-Based Molecular Networking (Nothias et al. 2020). A description on how this data was generated is provided in the GNPS Feature-Based Molecular Networking documentation. The data set consists of feature abundances (along with retention time and m/z values) and MS^2 spectra for these features. These example files are distributed through the github repository of the GNPS documentation https://github.com/CCMS-UCSD/GNPSDocumentation.git.

Below we first download the feature table (in csv format) from the above mentioned repository to a temporary file.

```
f_file <- "MZmine-GNPS_AG_test_featuretable.csv"
url <- paste0("https://raw.githubusercontent.com/CCMS-UCSD/GNPSDocumentation",
              "/master/docs/tutorials/AG_tutorial_files/")
curl_download(paste0(url, f_file),
              destfile = file.path(tempdir(), f_file))
```

We next load this file into R using the base `read.csv` function.

```
ftable <- read.csv(file.path(tempdir(), f_file), check.names = FALSE)
```

We can now use this file as an input for MS^1-based annotation, as shown in section Annotation using m/z values above, and match the m/z values of the features against HMDB assuming `[M+H]+` and `[M+Na]+` adducts. With parameter `mzColname` we define the column in the input file containing the m/z values. For MZmine this is column `"row m/z"`.

```
param <- Mass2MzParam(adducts = c("[M+H]+", "[M+Na]+"), ppm = 10)

f_match <- matchMz(
    ftable,
    compounds(cdb, c("compound_id", "exactmass", "formula", "name")),
    param = param,
    mzColname = "row m/z")
f_match
```

```
## Object of class Matched
## Total number of matches: 31333
## Number of query objects: 3443 (2251 matched)
## Number of target objects: 217776 (13194 matched)
```

For 2251 of the in total 3443 features a compound matching the feature's m/z was found.

MS^2 spectra can be exported from MZmine in mgf file format. Below we download the MGF file with all MS^2 spectra for the features from the example above to a temporary folder.

```
s_file <- "MZmine-GNPS_AG_test_GNPS.mgf"
curl_download(paste0(url, s_file),
              destfile = file.path(tempdir(), s_file))
```

Files in MGF format can be imported with the *MsBackendMgf* package. Below we use this package to create a `Spectra` object with all spectra from the example file.

```
library(MsBackendMgf)
sps_mgf <- Spectra(file.path(tempdir(), s_file),
                   source = MsBackendMgf())
```

By default all data fields from the original MGF file are imported. Thus, the `Spectra` object contains also a variable called `FEATURE_ID` with the identifiers for the features the spectra are associated with.

```
head(sps_mgf$FEATURE_ID)
```

```
## [1] "1" "2" "3" "4" "6" "7"
```

This `Spectra` object could then simply be used as in input parameter to the `matchSpectra` function as shown in the MS^2 annotation section above to e.g. identify matches with reference spectra from HMDB.

Package repositories and additional tutorials

- Tutorial with additional examples and explanations for MS^2-based annotations: https://jorainer.github.io/SpectraTutorials/
- Repository of the `MsCoreUtils` package: https://rformassspectrometry.github.io/MsCoreUtils/
- Repository of the `MetaboCoreUtils` package: https://rformassspectrometry.github.io/MetaboCoreUtils/
- Repository of the `Spectra` package: https://rformassspectrometry.github.io/Spectra/
- Repository of the `MetaboAnnotation` package: https://rformassspectrometry.github.io/MetaboAnnotation/
- Repository of the `CompoundDb` package: https://rformassspectrometry.github.io/CompoundDb/

Session information

The R version and versions of used packages are listed below.

```
sessionInfo()
```

```
## R version 4.1.2 (2021-11-01)
## Platform: x86_64-pc-linux-gnu (64-bit)
## Running under: Ubuntu 20.04.3 LTS
##
## Matrix products: default
## BLAS/LAPACK: /usr/lib/x86_64-linux-gnu/openblas-pthread/libopenblasp-r0.3.8.so
##
## locale:
##  [1] LC_CTYPE=en_US.UTF-8       LC_NUMERIC=C
##  [3] LC_TIME=en_US.UTF-8        LC_COLLATE=en_US.UTF-8
##  [5] LC_MONETARY=en_US.UTF-8    LC_MESSAGES=C
##  [7] LC_PAPER=en_US.UTF-8       LC_NAME=C
##  [9] LC_ADDRESS=C               LC_TELEPHONE=C
## [11] LC_MEASUREMENT=en_US.UTF-8 LC_IDENTIFICATION=C
##
## attached base packages:
## [1] stats4    stats     graphics  grDevices utils     datasets  methods
## [8] base
##
## other attached packages:
##  [1] MsBackendMgf_1.2.0      Spectra_1.5.7           MetaboCoreUtils_1.3.5
##  [4] pander_0.6.4            MetaboAnnotation_0.99.3 CompoundDb_0.99.5
##  [7] AnnotationFilter_1.18.0 curl_4.3.2              xcms_3.16.1
## [10] MSnbase_2.20.4          ProtGenerics_1.26.0     S4Vectors_0.32.3
## [13] mzR_2.28.0              Rcpp_1.0.8              Biobase_2.54.0
## [16] BiocGenerics_0.40.0     BiocParallel_1.28.3     knitr_1.37
## [19] BiocStyle_2.22.0        rmarkdown_2.11
##
## loaded via a namespace (and not attached):
##   [1] colorspace_2.0-2            rjson_0.2.21
##   [3] ellipsis_0.3.2              rprojroot_2.0.2
##   [5] XVector_0.34.0              fs_1.5.2
##   [7] GenomicRanges_1.46.1        base64enc_0.1-3
##   [9] clue_0.3-60                 remotes_2.4.2
##  [11] affyio_1.64.0               DT_0.20
##  [13] bit64_4.0.5                 fansi_1.0.2
```

```
##  [15] xml2_1.3.3               codetools_0.2-18
##  [17] ncdf4_1.19               doParallel_1.0.16
##  [19] cachem_1.0.6             impute_1.68.0
##  [21] robustbase_0.93-9        jsonlite_1.7.3
##  [23] dbplyr_2.1.1             cluster_2.1.2
##  [25] vsn_3.62.0               png_0.1-7
##  [27] BiocManager_1.30.16      compiler_4.1.2
##  [29] assertthat_0.2.1         Matrix_1.3-4
##  [31] fastmap_1.1.0            lazyeval_0.2.2
##  [33] limma_3.50.0             cli_3.1.1
##  [35] htmltools_0.5.2          prettyunits_1.1.1
##  [37] tools_4.1.2              gtable_0.3.0
##  [39] glue_1.6.1               GenomeInfoDbData_1.2.7
##  [41] affy_1.72.0              RANN_2.6.1
##  [43] dplyr_1.0.7              rsvg_2.1.2
##  [45] tinytex_0.36             MALDIquant_1.21
##  [47] vctrs_0.3.8              preprocessCore_1.56.0
##  [49] iterators_1.0.13         xfun_0.29
##  [51] stringr_1.4.0            ps_1.6.0
##  [53] lifecycle_1.0.1          XML_3.99-0.8
##  [55] DEoptimR_1.0-10          zlibbioc_1.40.0
##  [57] MASS_7.3-54              scales_1.1.1
##  [59] pcaMethods_1.86.0        MatrixGenerics_1.6.0
##  [61] parallel_4.1.2           SummarizedExperiment_1.24.0
##  [63] MassSpecWavelet_1.60.0   RColorBrewer_1.1-2
##  [65] yaml_2.2.2               memoise_2.0.1
##  [67] gridExtra_2.3            ggplot2_3.3.5
##  [69] MsFeatures_1.2.0         stringi_1.7.6
##  [71] RSQLite_2.2.9            highr_0.9
##  [73] foreach_1.5.1            pkgbuild_1.3.1
##  [75] GenomeInfoDb_1.30.1      rlang_1.0.0
##  [77] pkgconfig_2.0.3          matrixStats_0.61.0
##  [79] bitops_1.0-7             mzID_1.32.0
##  [81] evaluate_0.14            lattice_0.20-45
##  [83] purrr_0.3.4              ChemmineR_3.46.0
##  [85] htmlwidgets_1.5.4        bit_4.0.4
##  [87] processx_3.5.2           tidyselect_1.1.1
##  [89] plyr_1.8.6               magrittr_2.0.2
##  [91] R6_2.5.1                 IRanges_2.28.0
##  [93] generics_0.1.2           DelayedArray_0.20.0
##  [95] DBI_1.1.2                pillar_1.6.5
##  [97] withr_2.4.3              MsCoreUtils_1.6.0
##  [99] RCurl_1.98-1.5           tibble_3.1.6
## [101] crayon_1.4.2             utf8_1.2.2
## [103] grid_4.1.2               blob_1.2.2
## [105] callr_3.7.0              digest_0.6.29
## [107] munsell_0.5.0
```

References

Nothias, Louis-Félix, Daniel Petras, Robin Schmid, Kai Dührkop, Johannes Rainer, Abinesh Sarvepalli, Ivan Protsyuk, et al. 2020. "Feature-Based Molecular Networking in the GNPS Analysis Environment." *Nature Methods* 17 (9): 905–8. https://doi.org/10.1038/s41592-020-0933-6.

Stoffel, Rainer, Michael A. Quilliam, Normand Hardt, Anders Fridstrom, and Michael Witting. 2021. "N-Alkylpyridinium Sulfonates for Retention Time Indexing in Reversed-Phase-Liquid Chromatography-Mass Spectrometry–Based Metabolomics." *Analytical and Bioanalytical Chemistry*, December. https://doi.org/10.1007/s00216-021-03828-0.

Wishart, David S, AnChi Guo, Eponine Oler, Fei Wang, Afia Anjum, Harrison Peters, Raynard Dizon, et al. 2021. "HMDB 5.0: The Human Metabolome Database for 2022." *Nucleic Acids Research* 50 (D1): D622–31. https://doi.org/10.1093/nar/gkab1062.
